# New diastolic cardiomyopathy in patients with severe accidental hypothermia after ECMO rewarming: a case-series observational study

**DOI:** 10.1186/s12947-015-0027-2

**Published:** 2015-07-15

**Authors:** Tomasz Darocha, Dorota Sobczyk, Sylweriusz Kosiński, Anna Jarosz, Robert Gałązkowski, Krzysztof Nycz, Rafał Drwiła

**Affiliations:** Department of Anesthesiology and Intensive Care, The John Paul II Hospital, Medical College of Jagiellonian University, Cracow, Poland; Department of Interventional Cardiology, The John Paul II Hospital, Pradnicka 80, 31 202 Cracow, Poland; Department of Anesthesiology and Intensive Care, Pulmonary Hospital, Zakopane, Poland; Tatra Mountain Rescue Service, Zakopane, Poland; Department of Emergency Medical Services, Medical University of Warsaw, Polish Medical Air Rescue, Warsaw, Poland

**Keywords:** Deep accidental hypothermia, Echocardiography, Bi-ventricular diastolic dysfunction, Diastolic cardyomiopathy

## Abstract

**Introduction:**

Accidental hypothermia is a condition associated with significant morbidity and mortality. Hypothermia has been reported to affect left ventricular systolic and diastolic function. However, most of the data come from animal experimental studies.

**Aim of the study:**

The purpose of the present study was to assess the impact of severe accidental hypothermia on systolic and diastolic ventricular function in patients treated using veno-arterial extracorporeal membrane oxygenation (ECMO).

**Methods:**

We prospectively assessed nine hypothermic patients (8 male, age 25–78 years) who were transferred to the Severe Accidental Hypothermia Center and treated with ECMO. Transthoracic echocardiography was performed on admission (in patients without cardiac arrest) and on discharge from ICU after achieving cardiovascular stability. Cardiorespiratory stability and full neurologic recovery was achieved in all patients.

**Results:**

Biomarkers of myocardial damage (CK, CKMB, hsTnT) were significantly elevated in all study patients. Admission echocardiography performed in patients in sinus rhythm, revealed moderate-severe bi-ventricular systolic dysfunction and moderate bi-ventricular diastolic dysfunction. Discharge echocardiography showed persistent mild bi-ventricular diastolic dysfunction, although systolic function of both ventricles returned to normal. Discharge echocardiography in patients admitted with cardiac arrest showed normal (5 patients) or moderately impaired (1 patient) global LV systolic function on discharge. However, mild or moderate LV diastolic dysfunction was observed in all 6 patients. Discharge RV systolic function was normal, whereas mild RV diastolic dysfunction was present in these patients.

**Conclusion:**

After severe accidental hypothermia bi-ventricular diastolic dysfunction persists despite systolic function recovery in survivors treated with ECMO.

## Introduction

Accidental hypothermia is a condition associated with significant morbidity and mortality [1]. In the years 2009–2012 the Polish National Statistics Department reported 1836 deaths due to exposure to excessive natural cold [2]. In July 2013 the Severe Accidental Hypothermia Center was founded in the John Paul II Hospital in Cracow [3]. The Center is dedicated to invasive treatment patients from the Lesser Poland (Małopolska) province (area of 15 100 square km, population of 3.3 million) [3].

There are several reports describing the effects of mild therapeutic hypothermia on the circulatory system [4, 5] and only a few reports concerning severe hypothermia [6–8]. Hypothermia has been reported to affect left ventricular systolic and diastolic function [5, 7–9]. However, most of the data are derived from animal experimental studies [8–11]. To the best of our knowledge, this is the first study to describe the effects of severe accidental hypothermia and invasive ECMO rewarming on ventricular function in survivors.

### Study objective

The purpose of the present study was to assess the impact of severe accidental hypothermia on systolic and diastolic ventricular function in patients treated by means of veno-arterial extracorporeal membrane oxygenation (ECMO).

## Material and methods

The observational case-series study was approved by the Local Ethical Committee of the John Paul II Hospital in Cracow. After full recovery, all the study subjects gave informed consent to the use of their medical data.

We prospectively assessed 9 patients (8 male, age 25–78 years) who were reported to the Severe Accidental Hypothermia Center due to severe accidental hypothermia (core esophageal temperature 16.9-29 °C). All patients fulfilled the extracorporeal rewarming criteria and were admitted to our hospital for the implementation of invasive rewarming procedures.

A standard 12-lead electrocardiogram (ECG) was performed within 5 min after their admission to the Emergency Room in patients without cardiac arrest. Blood samples were collected for the plasma levels of biomarkers for myocardial damage: creatine kinase (CK), cardiac isoenzyme of creatine kinase (CKMB) and high sensitivity troponin T (hsTnT) at the time of admission and after 6 h. Blood tests were assayed by routine automated laboratory techniques (Cobas System 600, Roche Diagnostics GmbH, Manheim, Germany). All biochemical analyses were performed in the central hospital laboratory, certified with a cardiac and clinical chemistry program by RIQAS (Randox Quality Assessment Scheme, UK). Transthoracic echocardiography was performed on admission (in patients without cardiac arrest) and on discharge from the Intensive Care Unit (ICU) after achieving cardiovascular stability (in all patients). The examinations were conducted by means of a portable ultrasound system equipped with a 1–5 MHz transthoracic phased-array transducer (CX 50, Philips, Eindhoven, Netherlands). Comprehensive echocardiographic examination was performed by experienced cardiologists-echocardiographers. All the studies were recorded as digital 10-s video clips and independently analyzed by 2 echocardiographers. In each case a consensus was achieved, no ambiguous echocardiographic examinations were recorded and all results were clearly stated in a dichotomic manner.

The left ventricular ejection fraction (LVEF) was measured with the bi-plane Simpson method. Tricuspid annular plane systolic excursion (TAPSE) by M-mode was used to assess the global right ventricular systolic function. Pulsed-wave (PW) Doppler was performed in the apical 4-chamber view to obtain mitral and tricuspid inflow velocities. A sample volume was placed between the mitral or tricuspid leaflet tips during diastole. Spectral mitral and tricuspid velocity recordings were obtained at a sweep speed of 50 mm/s. PW tissue Doppler imaging (TDI) was performed in the apical views to acquire mitral and tricuspid annular velocities. The sample volume was positioned 1 cm within the septal and lateral insertion sites of the mitral leaflets and 1 cm within the lateral insertion site of the tricuspid leaflet. Spectral recordings were obtained at a sweep speed of 50 mm/s at end-expiration. Measurements obtained at 3 consecutive cardiac cycles were averaged [12, 13].

The following spectral Doppler (both mitral and tricuspid) parameters were taken into final analysis of left and right ventricular function: peak early filling (E-wave) velocity, late diastolic filling (A-wave) velocity, the E/A ratio, deceleration time (DT), early diastolic annular velocity (e’), the E/e’ ratio. Left ventricular (LV) diastolic dysfunction was diagnosed by the following TDI mitral parameters: septal e’ < 8 and lateral e’ < 10 cm/s [12]. In patients with mild diastolic dysfunction (grade 1), the mitral E/A ratio was 0.8, DT > 200 ms and average E/e’ ratio was < 8 [12]. Patients with moderate (grade 2) diastolic dysfunction had the following values: mitral E/A ratio 0.8-1.5, DT 160–200 ms, average E/e’ ratio 9-12 [12]. The following parameters indicated severe LV diastolic dysfunction: mitral E/A ratio ≥ 2, DT < 160 ms, average E/e’ > 13 [12]. Right ventricular (RV) diastolic dysfunction was graded according to the following guidelines: grade 1 (impaired relaxation) tricuspid E/A ratio < 0.8, grade 2 (pseudonormal filling) E/A ratio 0.8-2.1 and E/e’ ratio > 6, grade 3 (restrictive filling) E/A ratio > 2.1 with tricuspid DT < 120 ms [13].

## Results

The study population consisted of nine patients (eight male, age 25–78 years, average 51.3 ± 16.2 years), admitted to our hospital in severe accidental hypothermia (core temperature measured oesophagally 16.9-29 °C, average 23.6 ± 3.3 °C). Six patients were admitted in hypothermic cardiac arrest (4 with ventricular fibrillation, 2 with asystole) whereas 3 patients were in cardiogenic shock and their sinus rhythm was observed. Demographic and clinical data are presented in Table 1. All the patients fulfilled the extracorporeal rewarming criteria and in all of them veno-arterial extracorporeal membrane oxygenation (ECMO) was implanted. The duration of ECMO support was between 8 and 144 h (average 43.7 h, median 24 h). Cardiorespiratory stability and full neurologic recovery was achieved in all the patients (Glasgow Coma Scale 15, Cerebral Performance Category 1).

**Table 1:** Demographic and clinical characteristics of the study population

Nr	Sex/age (yrs)	Type of accident	Admission ECG	Core esophageal temperature (°C)	Hemodynamic status	Pre-rewarming catecholamine support	Duration of ECMO (h)	ICU duration (days)	Clinical status at discharge from ICU
1	M/29	Alpine	Asystole	22.3	Cardiac arrest	According to ERC 2010 resuscitation guidelines	32	8	Fully recovered
2	M/52	Urban	VF	22.2	Cardiac arrest		22	11	Fully recovered
3	M/78	Urban	VF	24	Cardiac arrest		27	17	Fully recovered
4	M/55	Urban	Asystole	24.1	Cardiac arrest		23	15	Fully recovered
5	F/25	Alpine	VF	16.9	Cardiac arrest		91	30	Fully recovered
6	M/60	Urban	VF	24	Cardiac arrest		8	12	Fully recovered
7	M/48	Urban	Sinus rhythm 60 bpm	24	Cardiogenic shock	Norepinephrine continuous infusion 5 ug/kg/h	22	54	Fully recovered
8	M/60	Urban	Sinus rhythm 60 bpm, atrio-ventricular block 1st degree	26.2	Cardiogenic shock	Dopamine continuous infusion 0.6 mg/kg/h	24	3	Fully recovered
						Norepinephrine continuous infusion 7.5 ug/kg/h			
9	M/55	Urban	Sinus rhythm 60 bpm	29	Cardiogenic shock	Dobutamine continuous infusion 0.5 mg/kg/h	144	16	Fully recovered
						Norepinephrine continuous infusion 5 ug/kg/h			

Biomarkers of myocardial damage (CK, CKMB, hsTnT) were significantly elevated in all the study patients (Table 2).

**Table 2:** Levels of biomarkers for myocardial damage measured on admission and after 6 h

Nr	On admission			6 h after admission		
	CK	CKMB	hsTnT	CK	CKMB	hsTnT
	(U/L)	(U/L)	(ng/ml)	(U/L)	(U/L)	(ng/ml)
1	87,191	564	0.671	103,812	558	0.489
2	329	113	0.204	607	108	0.575
3	1011	126	0.039	1603	125	0.616
4	1246	175	0.030	7344	254	0.816
5	2235	461	0.115	4138	164	2.51
6	5194	259	0.241	25,409	259	10.77
7	2397	115	0.027	2022	105	0.018
8	8794	441	0.029	6315	262	0.036
9	44,678	754	0.038	49,680	758	0.054

Normal values: CK 0–190 U/L, CKMB 0–24 U/L, hsTnT < 0.014 ng/ml

*CK* creatine kinase, *CKMB* cardiac isoensyme of creatine kinase, *hsTnT* high sensitivity troponin T

Admission echocardiography performed in patients in sinus rhythm, revealed moderate-severe bi-ventricular systolic dysfunction and moderate bi-ventricular diastolic dysfunction (Table 3). Discharge echocardiography in these 3 patients (nr 7–9) showed persistent mild bi-ventricular diastolic dysfunction although systolic function of both ventricles returned to normal (LVEF 50-65 %, TAPSE 20–23 mm).

**Table 3:** Baseline echocardiographic parameters in patients without cardiac arrest on admission

Nr	LVEF (%)	Mitral E/A	Mitral DT (ms)	LV E/e’	LV diastolic dysfunction	TAPSE (mm)	Tricuspid E/A	Tricuspid DT (ms)	RV E/e’	RV diastolic dysfunction
7	40	0.9	119	11	Grade 2	14	0.9	132	13.3	Grade 2
8	30	0.8	172	13	Grade 2	10	0.9	144	12.7	Grade 2
9	30	0.8	202	10	Grade 2	6	1.1	216	12	Grade 2

Five patients admitted with cardiac arrest had normal global LV systolic function on discharge. One patient (M/78 years) had moderately depressed LV systolic function (LVEF 40 %). All the patients had mild (50 % of patients) or moderate LV diastolic dysfunction. Discharge RV systolic function was normal, whereas mild RV diastolic dysfunction was present in all the six patients from this group (Table 4).

**Table 4:** Discharge echocardiographic parameters in all patients

Nr	LVEF (%)	Mitral E/A	Mitral DT (ms)	LV E/e’	LV diastolic dysfunction	TAPSE (mm)	Tricuspid E/A	Tricuspid DT (ms)	RV E/e’	RV diastolic dysfunction
1	65	1.3	162	9	Grade 2	22	0.7	221	5.5	Grade 1
2	40	0.7	193	7.2	Grade 1	20	0.8	150	4.8	Grade 1
3	60	0.9	164	9	Grade 2	21	0.6	172	4.3	Grade 1
4	55	0.7	211	11	Grade 1	25	0.7	112	3.2	Grade 1
5	60	1.3	126	9	Grade 2	20	0.7	166	2.5	Grade 1
6	60	0.7	175	6.2	Grade 1	30	0.6	261	4.5	Grade 1
7	50	0.6	121	5.8	Grade 1	20	0.8	117	2.9	Grade 1
8	60	0.7	198	7.2	Grade 1	22	0.6	202	3	Grade 1
9	65	0.6	200	6.2	Grade 1	23	0.7	250	3.6	Grade 1

## Discussion

Accidental hypothermia is defined as an unintentional drop in core temperature below 35 °C [1, 14]. Recent guidelines use a standard classification of hypothermia based on a core temperature and vital signs (Swiss staging system) [1, 14]. Severe hypothermia, defined as a core temperature of less than 28 °C, causes significant neurologic and circulatory disturbances [14].

There are several non-invasive and invasive rewarming techniques. However, choosing the appropriate rewarming method determines the long-term outcome [1]. Meta-analyses and long-term follow-up of severe hypothermia survivors validate the usefulness of extracorporeal membrane oxygenation (ECMO) in stages III and IV [14]. Early rewarming with ECMO has many advantages as compared with other rewarming methods [14]. That is why it is recommended by the European Resuscitation Council as the method of choice in rewarming from severe accidental hypothermia with cardiac instability or circulatory arrest [15]. All the patients in our study group fulfilled the extracorporeal rewarming criteria and in all of them arterio-venous extracorporeal membrane oxygenation (ECMO) was implanted.

Numerous animal studies have shown significant cardiac dysfunction caused by cooling and rewarming the intact heart in a non-arrested state [4–6, 8–11]. In the animal model, severity of hypothermia-induced cardiac dysfunction is dependent on the duration and depth of cooling [10]. The results of animal studies also suggest that hypothermia-induced cardiac dysfunction may be a decisive factor in the outcome from hypothermia and rewarming. Many researchers have tested the influence of hypothermia on mammalian heart tissue both in vitro and in vivo [4, 10, 11]. However, there are only several case reports regarding heart function during hypothermia in people [7]. Additionally, the long-term effects of hypothermia and rewarming on cardiac function have not yet been established. To the best of our knowledge, this is the first study to describe the effects of severe accidental hypothermia and invasive ECMO rewarming on the ventricular function in survivors.

Significantly elevated cardiac necrotic markers (CK, CKMB, TnT) were observed in all patients both on admission and 6 h later, indicating severe myocardial damage.

Baseline echocardiography (prior to ECMO implantation) was conducted in 3 patients in sinus rhythm on admission. The examination revealed moderate-severe bi-ventricular systolic dysfunction requiring inotropic support and moderate bi-ventricular diastolic dysfunction in these patients. Systolic LV and RV function returned to normal, whereas mild bi-ventricular diastolic dysfunction was still present on discharge. Discharge echocardiography in patients admitted with cardiac arrest showed normal (5 patients) or moderately impaired (1 patient) global LV systolic function. However, in all the 6 patients mild or moderate LV diastolic dysfunction was observed. Discharge RV systolic function was normal, whereas mild RV diastolic dysfunction was present in these patients.

There is no pre-hypothermia echocardiographic data to compare, because none of our patients had an echocardiographic study conducted before the incident. The follow-up period covered only ICU stay. However, despite these doubtless limitations, the data obtained allow the diagnosis of bi-ventricular diastolic cardiomyopathy in hypothermia survivors after ECMO rewarming persistent on discharge from the ICU. Mild LV diastolic dysfunction was present on discharge in six patients. Moderate LV diastolic dysfunction was observed in 3 patients: a 29-year-old male, a 25-year-old female and a 78-year-old male. Since age is a primary consideration when defining normal values of mitral inflow [12], all LV Doppler-derived parameters (mitral E/A, e’ and LV E/e’) were compared to normal values as specified by the guidelines. In all the patients LV diastolic echocardiographic parameters were reduced even when age-related changes were taken into account. Additionally, mild RV diastolic dysfunction was observed in the all patients on discharge.

Transient cardiac systolic and diastolic dysfunction was observed in subjects with mild therapeutic hypothermia [5] and in patients undergoing cardiac surgery with moderately hypothermic cardiopulmonary bypass [8]. Numerous animal experiments have investigated possible pathophysiological substrate of hypothermia-induced heart failure [9–11]. Studies in vitro indicated that calcium homeostasis and myofilament properties are affected by temperature, mostly via increasing the content of global cellular Ca^2+^ (caused by cold-induced alkalosis and inhibition of Na^+^/Ca^2+^ exchange) [9–11]. Tveita et al. [9, 11] reported a six to sevenfold increase in the calcium content in the hearts of re-warmed rats after 4 h of severe hypothermia. Wold et al. [10] found severe heart failure and significantly elevated cardiac Ca^2+^ after 4-h hypothermia in an animal model. Schwarzl et al. [4] concluded that mild hypothermia altered the diastolic function by inducing prolonged and incomplete LV relaxation despite spontaneous bradycardia in anesthetized pigs. However, the available experimental data do not fully explain the pathophysiological basis of post-hypothermic cardiomyopathy.

## Conclusion

Bi-ventricular diastolic dysfunction after severe accidental hypothermia persists despite the recovery of systolic function in survivors after ECMO rewarming.

### Key messages

- Severe accidental hypothermia causes significant bi-ventricular systolic and diastolic dysfunction.
- Ventricular systolic function returns to normal after ECMO rewarming.
- Bi-ventricular diastolic dysfunction after severe accidental hypothermia persists despite the recovery of systolic function after ECMO rewarming.

## Abbreviations

CKMB: Cardiac isoenzyme of creatine kinase
CPK: Creatine kinase
DT: Deceleration time
ECG: Electrocardiogram
ECMO: Arterio-venous extracorporeal membrane oxygenation
hsTnT: High sensitivity troponin T
ICU: Intensive care unit
LV: Left ventricle; left ventricular
LVEF: Left ventricular ejection fraction
PW: Pulsed-wave
RV: Right ventricle; right ventricular
TAPSE: Tricuspid annular plane systolic excursion
TDI: Tissue doppler imaging
